# Krüppel-like factor 5 remodels lipid metabolism in exercised skeletal muscle

**DOI:** 10.1016/j.molmet.2025.102154

**Published:** 2025-04-16

**Authors:** Konstantin Schneider-Heieck, Joaquín Pérez-Schindler, Jonas Blatter, Laura M. de Smalen, Wandrille Duchemin, Stefan A. Steurer, Bettina Karrer-Cardel, Danilo Ritz, Christoph Handschin

**Affiliations:** 1 Biozentrum, University of Basel, Basel, Switzerland; 2 sciCORE Center for Scientific Computing, University of Basel, Basel, Switzerland

**Keywords:** Skeletal muscle, Klf5, Exercise, Lipid metabolism, Stress response, Transcriptional regulation

## Abstract

Regular physical activity induces a variety of health benefits, preventing and counteracting diseases caused by a sedentary lifestyle. However, the molecular underpinnings of skeletal muscle plasticity in exercise remain poorly understood. We identified a role of the Krüppel-Like Factor 5 (Klf5) in this process, in particular in the regulation of lipid homeostasis. Surprisingly, gain- and loss-of-function studies in muscle *in vivo* revealed seemingly opposite functions of Klf5 in the response to an acute exercise bout and chronic training, modulating lipid oxidation and synthesis, respectively. Thus, even though only transiently induced, the function of Klf5 is complex and fundamental for a normal long-term training response. These findings highlight the importance of this mediator of external stress response to adaptive remodeling of skeletal muscle tissue.

## Highlights

• Klf5 is transiently induced and activated by an acute exercise bout in muscle.
• In an acute bout, Klf5 regulates fatty acid metabolism in skeletal muscle.
• In chronic training, Klf5 controls lipid biosynthesis, including of cardiolipin.
• Without Klf5, training leads to the accumulation of potentially toxic lipid species.
• Klf5 is indispensable for a normal training adaptation.

## Graphical abstract

**Figure:**
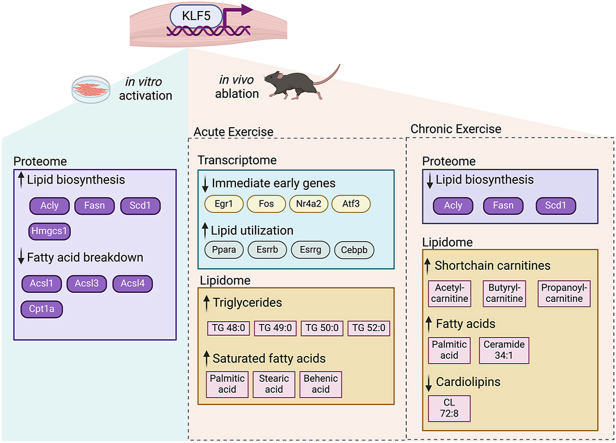

## 1 Introduction

Regular physical activity is a cornerstone of health and longevity. Accordingly, exercise not only increases physical capacity, but also exerts numerous physiological benefits that extend beyond skeletal muscle [1]. For example, improved cardiovascular health and enhanced metabolic efficiency evoked by training can counteract many of the pathological events in non-communicable diseases such as type 2 diabetes, hypertension or heart disease [2,3]. Understanding the molecular mechanisms underpinning exercise-mediated adaptations in skeletal muscle thus could help to identify targets that might be leveraged for health promotion or disease prevention in people unable to exercise.

Skeletal muscle exhibits a high degree of plasticity and can adapt and remodel depending on the stimulus provided during exercise training. At the moment, it is unclear how the transient response to an acute exercise bout is integrated and interpreted to lead to persistent training adaptations if exercise is repeated over weeks and months [1,2]. For example, the perturbations that are elicited by an acute exercise bout evoke a general stress response, in particular in training-naïve muscle, that is similar to the events observed in other cell types, tissues and organs in comparable settings [4,5]. With repeated bouts, a higher specification of the response might be attained [1,2]. The engagement of these initial signaling pathways, transcription factors, and the ensuing immediate- and intermediate-early stress response gene expression mitigates cellular stress, induces counteracting pathways aimed at restoration of homeostasis, and initiates adaptive processes to increase resilience to future insults of the same kind [6]. Of note, these pathways can be pathological or protective, most likely depending on the prevailing context and environment [7]. Intriguingly, the role and tissue-specificity of this stress response in the response of skeletal muscle to an acute exercise bout and chronic training adaptation is unclear. While pulsatile activation of parts of this stress-response in has been shown to mimic some of the exercise-induced benefits, it is unclear how the exact adaptations are orchestrated and the transcriptional network following exercise is regulated [8]. To address some of these open questions, we have therefore studied the contribution of the transcription factor Krüppel-Like Factor 5 (Klf5) to exercise adaptations in mouse skeletal muscle. Klf5 is expressed in various tissues and has been linked to stress contexts in a number of pathologies, being activated by the MEK-ERK1/2- early growth response-1 (EGR-1) immediate early gene pathway amongst others [9]. The role of Klf5 in skeletal muscle is complex, with potential functions spanning from regeneration and atrophy to lipid metabolism [[6], [7], [8]]. In the context of muscle regeneration, Klf5 expression is upregulated in differentiating myocytes and in centrally located nuclei following injury. Accordingly, genetic ablation in satellite cells results in impaired skeletal muscle regeneration and fibrosis [10]. Second, increased Klf5 expression is observed in various atrophic conditions such as microgravity, dexamethasone treatment, hind limb suspension and sarcopenia [11]. Inhibition of Klf5 alleviates muscle atrophy by reducing expression of the atrogenes Fbxo32 and Trim63 in synergy with Foxo1 [11]. Finally, Klf5 is induced upon a high-fat diet in the *soleus* muscle of wild-type mice, and its heterozygous gene ablation increases energy expenditure and fatty acid oxidation, mediated by diminished Pparδ repression [12]. Besides this regulation and function in pathological contexts, increased Klf5 activity has been predicted in skeletal muscle after an acute, maximal endurance exercise bout in both control and high fat diet-fed mice [13]. It thus is unclear whether Klf5 regulation and function in skeletal muscle is confined to perturbations elicited by pathological stressors, or if this transcription factor also contributes to physiological training adaptation. In this study, we leveraged viral vector-mediated knockdown (KD) and overexpression (OE) of Klf5 *in vitro* and *in vivo* to study this question, and now provide evidence for an important role of Klf5 as a regulator of endurance-exercise adaptation both in the acute exercise as well as chronic training paradigms.

## 2 Material and methods

### 2.1 Animal experiments

Male C57BL/6J mice between the ages of 8–24 weeks were kept under standard conditions with a 12/12-hour light/dark cycle and access to food and water *ad libitum*. All experiments were approved by the Kantonales Veterinäramt Basel-Stadt and followed the Swiss guidelines for animal experimentation and care.

### 2.2 Electric pulse stimulation (EPS) in C2C12 myotubes

C2C12 myoblasts were seeded at a density of 300k cells/well in proliferation medium (DMEM high glucose (Sigma) with 10% fetal bovine serum) until confluency was reached. Subsequently, confluent myoblasts were differentiated into myotubes in differentiation medium (DMEM high glucose (Sigma) with 2% horse serum) for 72h. Medium was exchanged directly before EPS treatment. EPS was performed for 2h at 30V, 2 ms, 5Hz. Cells were harvested immediately or 1h post-EPS.

### 2.3 Metabolic phenotyping of C2C12 myotubes (Seahorse XF96 analyzer)

C2C12 cells were seeded at 10′000 cells per well, cultured to confluence, and differentiated in 2% horse serum for 72h. Seahorse Mito Stress and Glycolytic Rate Assays were performed using the Seahorse XF96 Analyzer, measuring oxygen consumption rate (OCR) and extracellular acidification rate (ECAR) following the manufacturer's instructions. Normalization of data was performed based on fluorescence intensity from DAPI staining (1:1000 dilution) conducted following completion of the respective assay. Data were analyzed using Seahorse Wave software.

### 2.4 Integration of ChIP-seq data

ChIP-seq peaks from publicly available datasets [10] of Klf5 (GSE80812) in C2C12 myotubes were visualized on the Integrated Genome Browser-13.2 to generate representative genome browser figures. ChIP-seq peaks were associated to genes by ChIPSeeker v1.32.1 [14].

### 2.5 Intravenous (i.v.) AAV application

C57BL/6J mice were injected via the lateral tail vein at the age of 8 weeks with 10^12^vg per mouse in a volume of 100 μl PBS. After injection, mice had an incubation time for 3 weeks during which no further tests were performed. Mice were injected with AAVMyo-U6-Klf5-shRNA or AAVMyo-U6-SCR-shRNA for the systemic knockdown or AAVMyo-dMCK-Klf5-miRNA or AAVMyo-dMCK-eGFP-miRNA, respectively.

### 2.6 Intramuscular (i.m.) AAV application

C57BL/6J at the age of 16 weeks were injected with 10^11^vg per gastrocnemius muscle. Mice were anesthetized using inhalation anesthesia (isoflurane 3%, carrying gas O2, 400–600 ml/min). One leg was injected with AAV9-Klf5-shRNA while the contralateral leg was injected with control AAV9-SCR-shRNA.

### 2.7 AAV production

AAVs were produced following the protocol described previously [15]. Briefly, 20 × 15 cm dishes of confluent HEK293T cells cultured in DMEM high glucose (Sigma) with 10% FBS were triple transfected with AAV-plasmid (shRNA targeted against Klf5 (GCGATTCACAACCCAAATTTA), miRNA targeted against Klf5 (TTCCGATAATTTCAGAGCATAA, TGCCGCTACAATTGCTTCCAAA) or Scrambled control purchased from Vectorbuilder), AAV9 (a gift from James M. Wilson, Addgene plasmid # 112865) or AAVMYO plasmid (a gift from Dirk Grimm) and pAdDeltaF6 helper plasmid (a gift from J.M. Wilson, Addgene#112867; purchased from PlasmidFactory GmbH). Medium was collected 72h and 120h post-transfection and incubated with 8% PEG8000 for AAV-particle precipitation for 2h at 4 °C, then spun down at 4 °C at 4000g for 30min. The resulting pellet was resuspended in AAV-lysis buffer (50 mM Tris-HCl, 1M NaCl, 10 mM MgCl_2_ with 50U salt activated nuclease (SAN)). Cells were harvested 120h post-transfection, pelleted for 10min at 500g and lysed in 5 ml AAV-lysis buffer. Both lysates were pooled and incubated for 1h at 37 °C prior to purification. AAV particles were purified via Iodixanol-gradient (StemCell) purification (15%, 25%, 40%, 60%), concentrating in the 40% layer. Afterwards, the 40% layer was passed through a 100kD MWCO filter (Amicon) and washed with PBS for 4 times. Tittering was performed using RT-PCR against a standard curve consisting of a PvuI digested ITR-containing plasmid as described [16].

### 2.8 Adenoviral vector production

Adenovirus vectors were generated using the Adeno-X Adenoviral System 3 according to the manufacturer's protocol (Takara, #632267). The Klf5 sequence and the LacZ gene was subcloned into the pAdenoX-ZsGreen1 vector, which was subsequently used to generate a LacZ control adenovirus. All plasmids were validated through Sanger sequencing. Adenovirus production and amplification were carried out in Adeno-X 293 cells (Takara, #632271), and viral titers were quantified by fluorescence-activated cell sorting (FACS).

### 2.9 Adenoviral transduction

Differentiated C2C12 myotubes were exposed to adenovirus encoding HA-Klf5 or LacZ adenovirus, with a multiplicity of infection MOI = 6. Cells were transduced for 4 h before rinsing once using phosphate-buffered saline (PBS), and subsequent cultivation in a medium devoid of adenovirus for an overall period of 48 h.

### 2.10 Maximum capacity test

Exhaustive treadmill tests were performed using two protocols. Prior to the test, all mice were acclimatized to the treadmill on two days (day 1: 0 m/min, 5 m/min, 10 m/min each for 5min, day 2: 5 m/min, 10 m/min, 12 m/min each for 5min). Mice injected i.v. were subjected to the test on a closed treadmill (metabolic modular, Columbus instruments) at a 5° incline with the following protocol: starting at 8 m/min for 3 min and subsequent increase in speed by 2 m/min every 3min up to 38 m/min. After that, the speed was increased every 10min by 2 m/min until exhaustion was reached. Maximum capacity tests for i.m. injected mice were performed with the same increase in speed, starting at 8 m/min for 3min and afterwards the speed increased 2 m/min every 3min until exhaustion at 5° slope on an open treadmill (Columbus Instruments).

### 2.11 Metabolic phenotyping

Metabolic phenotyping was performed using the comprehensive laboratory animal monitoring systems (CLAMS, Columbus Instruments). Mice were housed for 96h within a sealed cage at 23 °C with a 12h light/dark cycle. During this time activity, food consumption, weight, oxygen consumption and CO_2_ exhaustion were measured every 15min.

### 2.12 RNA Extraction and RT-PCR

Total RNA was extracted from pulverized muscle tissue. 20 mg of tissue were dissolved in 1 ml TRIreagent (Sigma) and lysed using an MP-Tissue Lyser (3 cycles for 10s at 5 m/s) and FastPrep Tubes (MP Biomedicals). After lysis, 200 μl of chloroform were added to the lysate, followed by a centrifugation for 15min at 13000g. Next, the aqueous phase was mixed 1:1 with 100% Ethanol and purified using the Direct-zol RNA Purification Kit (Zymo) according to manufacturer's instructions. RNA concentration was measured using a NanoDrop OneC spectrophotometer (Thermo Scientific) and 1 μg of RNA was subsequently used for cDNA synthesis by reverse transcription with the High-Capacity cDNA RT Kit (Applied Biosystems). qPCR was performed using Fast SYBR Green (Applied Biosystems) with primers listed in table 1.

**Table 1:** List of qPCR primers.

Gene	Sequence Forward	Sequence Reverse
Klf5	GCAGAATCTCACCCCACCTC	TCGCAGAAGTGGATACGTCG
Scd1	TTCTTGCGATACACTCTGGTGC	CGGGATTGAATGTTCTTGTCGT
Acly	ATCAACCCCCTTGTGGTGA	GCTTCAAGCTTGCTCCACTT
Acaca	TGTCCGCACTGACTGTAACC	ATTTCCATAGCCGACTTCCA
Fasn	GCTGCGGAAACTTCAGGAAAT	AGAGACGTGTCACTCCTGGACTT
Tbp	TGCTGTTGGTGATTGTTGGT	CTGGCTTGTGTGGGAAAGAT

### 2.13 Protein extraction and immunoblotting

Pulverized muscle tissue (30 mg) was homogenized in 300 μl of protein lysis buffer (50 mM Tris-HCl, 150 mM NaCl, 1 mM EDTA, 5% Glycerol, 1% NP40, 0.1% SDS) using an MP-Tissue Lyser (3 cycles, 6 m/s for 10s, 60s pause). The homogenate was incubated at 4 °C for 30 min while shaking, then centrifuged at 12,000g for 10 min at 4 °C. Protein concentration in the supernatant was quantified using Bradford protein assay. Samples were normalized and mixed with Laemmli sample buffer and heated to 95 °C for 10min. Proteins were separated via SDS-PAGE and transferred onto a nitrocellulose membrane. The membrane was blocked with 5% non-fat milk in TBST for 1 h at room temperature, followed by overnight incubation at 4 °C with the primary antibody (Klf5 (D7S3F), CST; Vinculin (7F9), SC). HRP-linked secondary antibody was incubated for 1 h at RT. Proteins were detected using the Femto Maximum Sensitivity Substrate (Thermo Scientific) by chemoluminescence.

### 2.14 RNA-Seq analysis

17-week old C57BL/6JRj mice were injected with AAV9-U6-Klf5 shRNA in the right leg and AAV9-U6-Scr shRNA in the contralateral leg as control. After 4 weeks of incubation time, mice were subjected to a maximum capacity test and sacrificied 3h post-exercise. RNA extraction was performed using *Zymo* RNA-Extraction kit and sequencing was done on a Novaseq6000 using a S1 PE60/62 flow cell. Raw reads were trimmed with trimmomatic [17] (paired-end mode, PHRED quality threshold of 15 for leading and trailing bases, 4-base wide sliding window with a PHRED threshold of 20). Raw and trimmed reads were then aligned using STAR [18] on the Mouse genome (GRCm39) with gene annotations from Ensembl 103. Reads and alignment quality was assessed using fastqc and multiqc [19], and we retained the trimmed reads for subsequent analysis as they yielded a higher number of uniquely mapped reads. Gene expression levels were assessed from the alignment by performing stranding-aware count of uniquely mapped reads (STAR -geneCount option). Differential Expression analysis was conducted using the DESeq2 [20] library, with a filter for genes with least 10 reads in at least 4 samples, and using a model accounting for exercise level (sedentary, exercised), and its interaction with individuals (as each mice were subject to 2 measurements) and type of injection (KLF5 knock-down or scramble). The ashr library was used to obtain shrunk log-Fold-changes [21].

### 2.15 Voluntary wheel running (VWR)

As an endurance training modality, mice were given access to running wheels for 6 weeks. Running wheel activity was monitored throughout the time of the study. To confirm a trained phenotype, maximum capacity tests were performed in the 5th week of access to running wheels. Running wheels were blocked 36h before dissection of the mice to avoid confounding effects of the last acute exercise bout.

### 2.16 TMT labelling and LC MS/MS analysis

Proteomics were performed as described previously [22]. Sample aliquots containing 18 μg (TMT10plex) or 10 μg (TMTpro16) of peptides were labeled with TMT reagents (Thermo Fisher Scientific) as previously described [23]. Peptides were resuspended in labeling buffer (2M urea, 0.2M HEPES, pH 8.3) and labeled with TMT reagents followed by a 1h incubation at 25 °C. To control ratio distortion, a peptide calibration mixture was added before labeling with TMT10plex reagents. Aqueous 1.5M hydroxylamine solution was added to quench labeling followed by pooling of samples. The pH was adjusted to 11.9 and incubated to remove TMT labels. The reaction was stopped with 2M hydrochloric acid until a pH < 2 was reached. Peptide samples were desalted, fractionated, and dried. LC–MS/MS analysis was performed using a Q Exactive HF Mass Spectrometer. Peptides were resolved using RP-HPLC. Mass spectrometry was operated in DDA mode. Spectra were searched against a murine database using SpectroMine software. Acquired reporter ion intensities were used for quantification and statistical analysis using SafeQuant R script (v2.3). Data were normalized, transformed, and analyzed for differential abundance using empirical Bayes moderated t-statistics.

### 2.17 Gene ontology analysis

Gene ontology (GO) analysis was performed with gene lists derived from RNA-Seq and proteomics via the Database for Annotation, Visualization, and Integrated Discovery (DAVID v2022q2). Significance was based on a Bonferroni corrected P-Value of less than 0.05.

### 2.18 Lipidomics

Lipid extraction was performed as described previously [24] with some modifications. After brief vortexing of the homogenates, the samples were continuously mixed in a thermomixer (Eppendorf) at 25 °C (950rpm, 30min). Protein precipitation was obtained after centrifugation for 10min at 16000g and 25 °C. The single-phase supernatant was collected, dried under N_2_, and stored at −20 °C until analysis. Before analysis, the dried lipids were redissolved in 100 μl Methanol:Isopropanol (1:1). Liquid chromatography was done as described previously [25,26] with some modifications. A binary UPLC pump (Vanquish, Thermo Scientific, Germany) was used with the following mobile phases; A) Acetonitrile: H_2_O (6:4) with10mM ammonium acetate and 0.1% formic acid and B) Isopropanol: Acetonitrile (9:1) with 10 mM ammonium acetate and 0.1% formic acid. The lipids were separated using the Acquity BEH C18 column (Waters) with the dimensions 30 mm × 2.1 mm × 1.7 μm (length × internal diameter × particle diameter). The column temperature was set to 60 °C. The following gradient was used with a flow rate of 1.2 ml/min; 0.0–0.29min (isocratic 15–30%B), 0.29–0.37min (ramp 30–48% B), 0.37–1.64min (ramp 48–82%B),1.64–1.72min (ramp 82–99%), 1.72–1.79min (isocratic 99%B), 1.79–1.81min (ramp 100-15% B) and 1.81–2.24min (isocratic 15%B). Injection volume was 2 μl. The needle wash solvent was Methanol: Isopropanol: Acetonitrile: H_2_O (1:1:1:1). The liquid chromatography was coupled to a hybrid quadrupole-orbitrap mass spectrometer (Q-Exactive HFx, Thermo Scientific, Germany). Heated electrospray ionization was used with the following source parameters: sheath gas flow rate 40, aux gas flow rate 8, spray voltage 3.5 kV, capillary temperature 300 °C, funnel RF level 50, and auxiliary gas heater temperature 300 °C. The mass spectrometer was operated in data-dependent acquisition mode DDA). First, a full scan was used scanning from 200 to 2000m/z at a resolution of 60000 and AGC Target 1e6, max injection time 100 ms. Then the top two precursors were automatically selected for fragmentation using normalized collision energies (NCE) of 20, 30,50na resolution of 7500, and an AGC target of 1e5. Raw mass spectrometric data were imported in Compound Discoverer 3.3 (ThermoScientific) for data analysis. Peak picking, retention time alignment, and compound grouping were performed; Afterwards, lipid annotation was performed by matching the MS2 spectra to the LipidBlast in-silico library. Lipid identification was manually confirmed based on LSI criteria and annotations not matching the stringent criteria were filtered out. Peak areas were normalized using median normalization and differential analysis was performed between the different groups and the p-values were corrected for multiple comparisons.

### 2.19 Statistical analysis

The transcriptomic, lipidomic, and proteomic data were statistically analyzed according to the methods detailed in their respective sections. Additional statistical tests were conducted using GraphPad Prism v.9, applying either a two-tailed Student's t-test or a two-way analysis of variance (ANOVA) with Šídák's multiple-comparison test. Results are presented as mean ± s.e.m., and a significance level of P < 0.05 was generally considered statistically significant.

## 3 Results

### 3.1 Expression and motif enrichment of Klf5 increase after an acute bout of exercise

**Figure 1:**
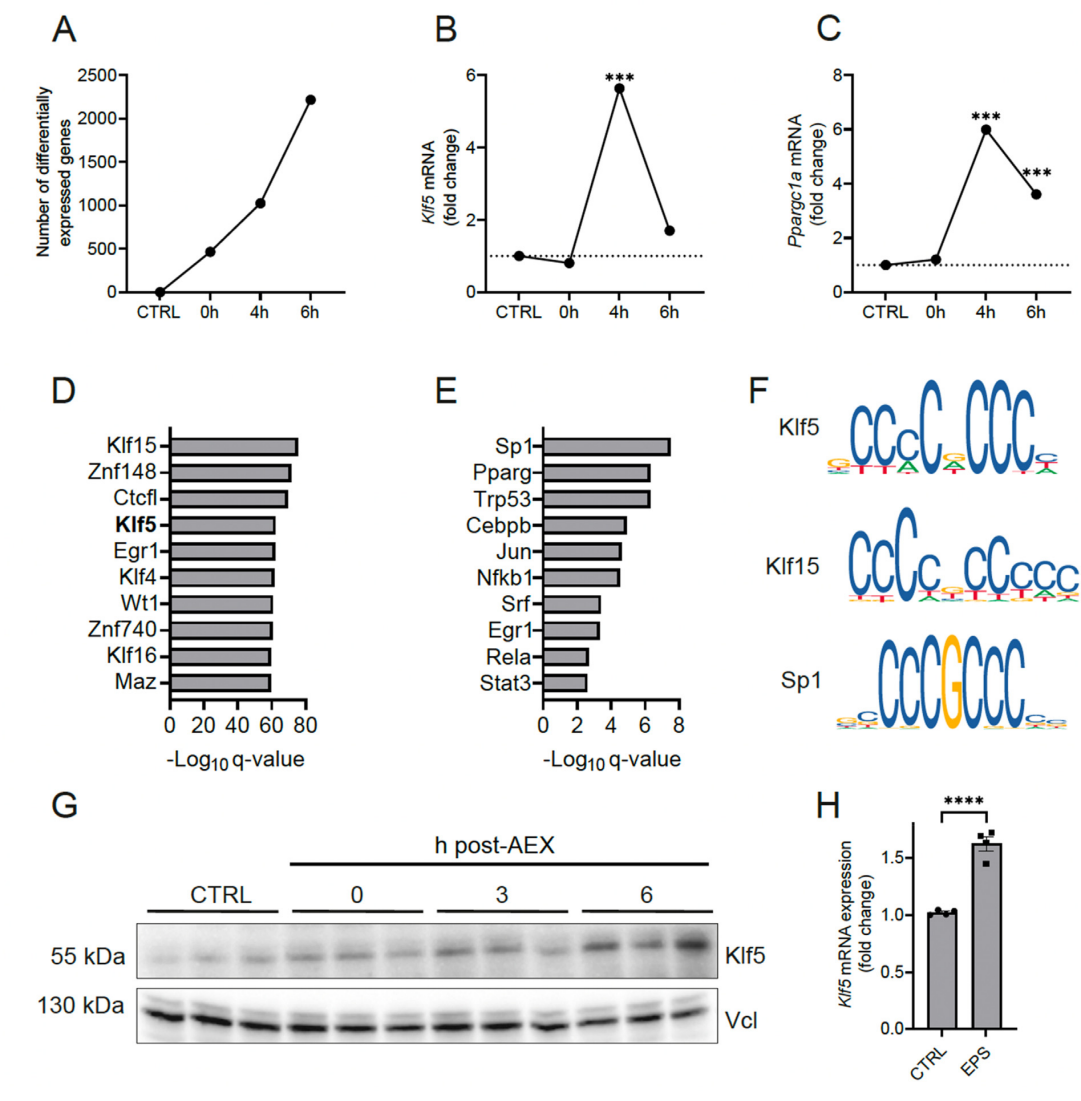
**Expression and motif enrichment of Klf5 increase after an acute bout of exercise.** **A)** Number of differentially expressed genes at 0h, 4h and 6h post-exercise. **B)***Klf5* expression post-exercise. **C)***Ppargc1a* (encoding PGC-1α) expression post-exercise. **D)** Transcription factor activity prediction of upregulated genes at 6h post-exercise (pScan). **E)** Transcription factor activity prediction of upregulated genes at 6h post-exercise (TTRUST). **F)** Klf5, Klf15, Sp1 consensus sequences (JASPAR), **G)** Klf5 protein levels in control muscles and at different time points post-exercise. **H)***Klf5* mRNA expression in C2C12 myotubes 1 h after EPS treatment. Statistical test: student's t-test, corrected for multiple comparisons -significant at the 4h time point, student's t-test: H; ∗p < 0.05, ∗∗p < 0.01, ∗∗∗p < 0.001. Data represented as mean ± SEM.

Skeletal muscle reacts to an acute exercise stimulus with the unfolding of a transcriptomic network indicated by the increase of differentially expressed genes in the hours post-exercise (Figure 1A). Expression of Klf5 transiently increases 4h after activity cessation (Figure 1B), showing a similar expression profile as the peroxisome proliferator-activated receptor γ coactivator 1α (Pgc-1α) (Figure 1C), a well-established regulator of exercise-adaptation (data shown from published dataset accessible at https://myo-trex.scicore.unibas.ch/) [[25], [26], [27], [28], [29], [30]]. To assess how changes in transcript levels translate into function, Klf5 activity was predicted by motif enrichment analysis leveraging two different methods, pScan and TTRUST [31,32]. pScan identifies the Klf5 motif as one of the top five enriched motifs with Klf15 ranking as the highest based on the gene expression changes 6h post-exercise (Figure 1D). Notably, both Klf5 and Klf15 share high similarities in consensus binding motifs, but in contrast to Klf5, Klf15 was not differentially expressed 4h and 6h post-exercise. Furthermore, while the TTRUST algorithm predicted the Sp1 motif as the top-target, implying activation of this transcription factor that, similar to the Klfs, binds to GC-box consensus sequences (Figure 1E), Sp1 was actively downregulated post-exercise (data not shown). Thus, while contributions of Klf15 and Sp1 cannot be excluded, it is possible that the strong enrichment of GC-rich motifs could be mainly attributed to Klf5. Importantly, in line with the transcriptional induction at 4h, and the predicted increase in motif enrichment at 6h, Klf5 protein levels were elevated 6h post-exercise (Figure 1G). Since the transcriptional and protein data were derived from bulk muscle samples including different cell types besides myofibers, we examined whether Klf5 is regulated in muscle cells by assessing the effect of EPS treatment on mouse C2C12 myotubes. In this experimental contexts, elevated Klf5 transcript levels were found after EPS, indicating that, at least in part, the exercise-mediated induction of Klf5 is occurring in muscle fibers (Figure 1H).

### 3.2 Short-term overexpression of Klf5 regulates proteins involved in fatty acid biosynthesis in C2C12 myotubes

**Figure 2:**
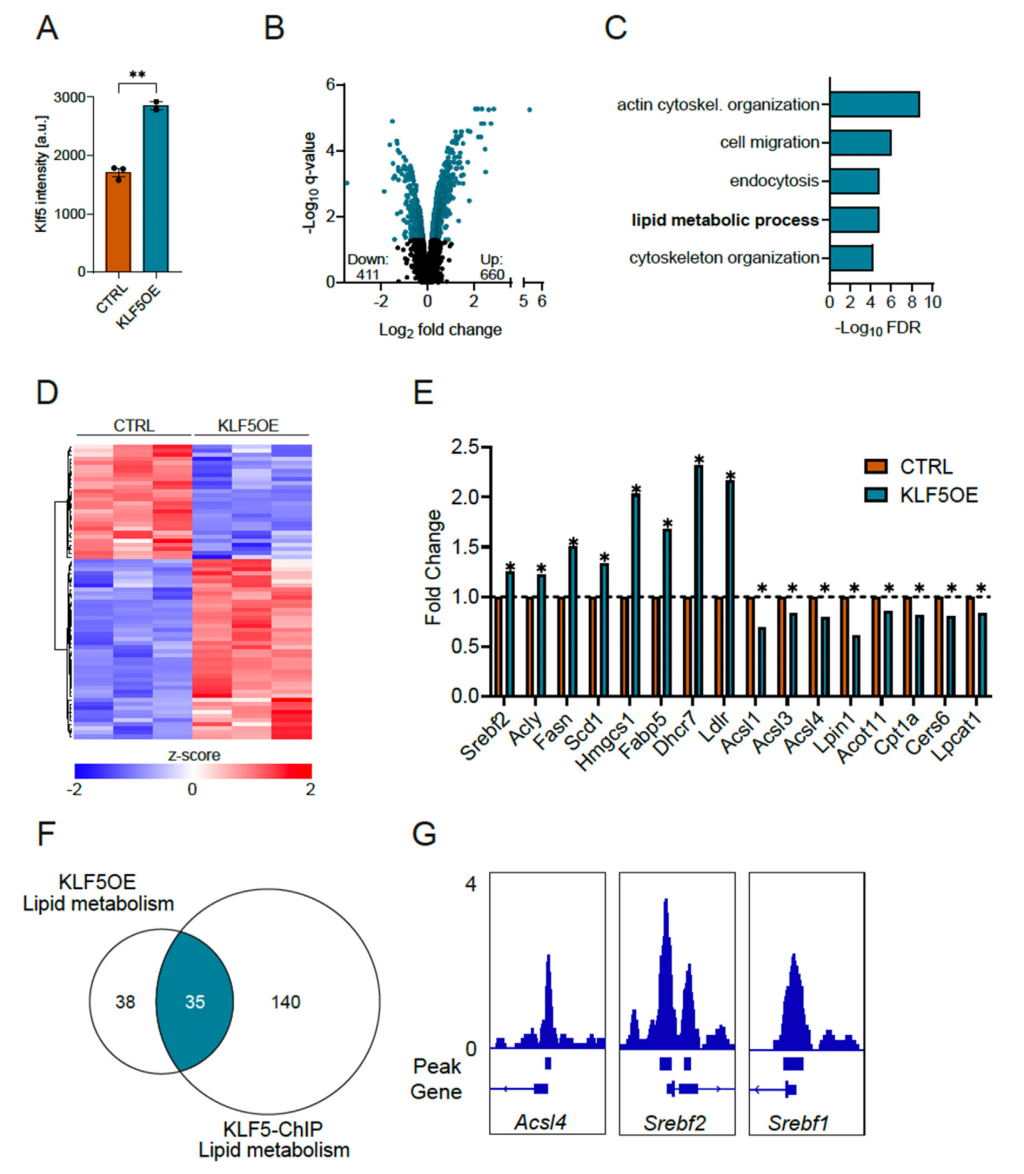
**Short-term overexpression of Klf5 regulates proteins involved in fatty acid biosynthesis in C2C12 myotubes.** **A)** Protein abundance of Klf5 (a.u. arbitrary units, n = 3, 3). **B)** Differentially expressed proteins in Klf5 overexpression (OE) compared to control myotubes (q-value<0.05, FC > 1.1). **C)** Top 5 terms of GO-analysis (biological processes) of differentially expressed proteins. **D)** Heat map of the proteins differentially regulated and involved in lipid metabolism. **E)** Selection of differentially detected proteins involved in lipid metabolism. **F)** Overlap between proteins found in the Klf5OE and Klf5-ChIP-Seq in the GO-Term “Lipid metabolism”. **G)** Representative significant peaks for the genes *Acsl4*, *Srebf2* and *Srebf1*. Statistical test: student's t-test: A; Bayes moderated t-statistics (proteomics data): E. ∗p < 0.05, ∗∗p < 0.01, ∗∗∗p < 0.001. Data represented as mean ± SEM.

Based on the acute transient expression of Klf5 post-exercise, we sought to explore the impact of short-term overexpression of Klf5 on muscle function. We therefore performed proteome analysis of C2C12 myotubes transduced for two days with an adenoviral vector encoding mouse Klf5. As expected, Klf5 protein levels were 2.6-fold increased (Figure 2A) and resulted in 660 upregulated and 411 downregulated proteins (q-value>0.05, FC > 1.1) (Figure 2B). Gene ontology enrichment analysis revealed multiple terms including “actin cytoskeleton organization”, “cell migration” and “lipid metabolic process” (Figure 2C, only top five terms are shown, overall 38 terms were significantly predicted when using a Benjamini-Hochberg p-value<0.05). Modifications of lipid metabolism are an integral part of exercise adaptation [33]. Intriguingly, Klf5 overexpression evoked a distinct pattern (Figure 1D) leading to downregulation of proteins involved in fatty acid breakdown while inducing proteins associated with fatty acid biosynthesis, among them Srebf2, a master regulator of lipid anabolism and the biosynthetic genes Acly, Fasn and Scd1 (Figure 1E). To discern primary from secondary Klf5 target genes, we made use of a publicly accessible ChIP dataset of endogenous Klf5 in C2C12 myotubes [10]. Focusing on genes/proteins related to lipid metabolism from both datasets revealed a common set of 35 genes/proteins (Figure 2F). In line with the proposed function of Klf5 as an transcriptional activator or repressor [12], we observed chromatin recruitment of Klf5 in the vicinity of upregulated, e.g. Srebf2, and downregulated genes, e.g. Acsl4. The presence of Klf5 binding peaks in additional genes for which no protein was detected in the mass spectrometry, e.g. Srebf1, implies an even broader role of this transcription factor in the control of fatty acid homeostasis (Figure 2G). To further evaluate the potential effects of KLF5 overexpression on cellular metabolism, we conducted mitochondrial stress tests and glycolytic rate assays. Overexpression of KLF5 did not alter oxygen consumption during the mitochondrial stress test, indicating no impact on mitochondrial function under either basal conditions or following FCCP-induced stimulation (Figure S1A and B). In contrast, glycolytic rate measurements revealed an increase in both the basal extracellular acidification rate (ECAR) and the basal glycolytic proton efflux rate (glycoPER) upon KLF5 overexpression (Figure S1C and D), suggesting a shift towards enhanced glucose metabolism under these conditions.

### 3.3 Muscle specific knockdown of Klf5 reduces voluntary activity and aerobic metabolism during exercise

**Figure 3:**
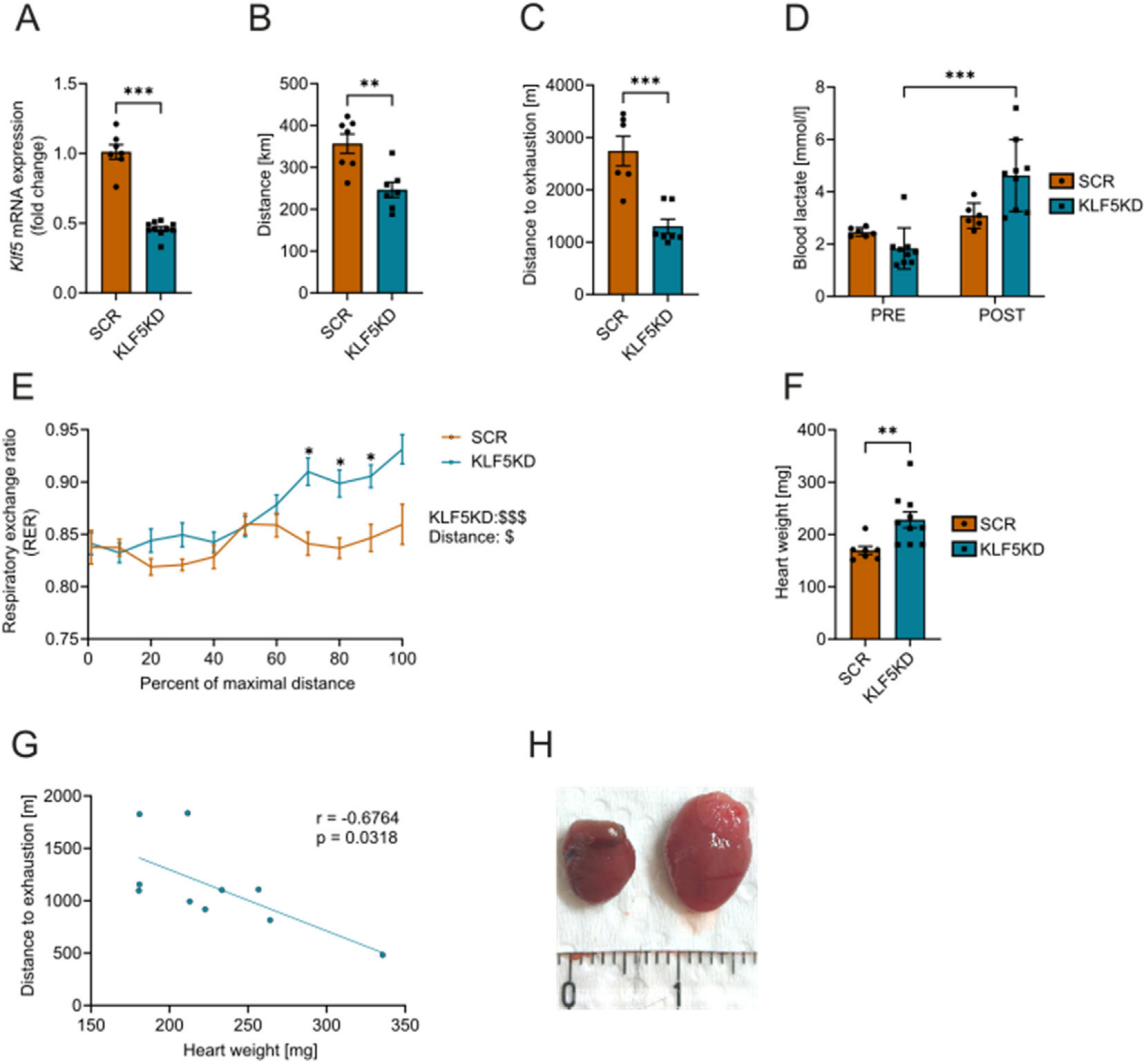
**Striated muscle specific knockdown of *Klf5* reduces voluntary activity and aerobic metabolism during exercise in the context of cardiac hypertrophy.** **A)** Relative *Klf5* mRNA expression in GAS. **B)** Total running distance covered in 6 weeks of access to running wheels. **C)** Distance to exhaustion in a maximum capacity test on a treadmill. **D)** Blood lactate pre- and post-exercise, **E)** Respiratory exchange ratio during maximum capacity test. **F)** Heart weight after dissection. **G)** Pearson correlation of distance to exhaustion versus heart weight. **H)** Representative pictures of the heart of SCR-animals (left) and KLF5KD (right). For all experiments n= (7, 9). Statistical test: two-way ANOVA (repeated measures) followed by Sidak's multiple comparisons 2 groups: D, E; student's t-test: A, B, C, F. ∗p < 0.05, ∗∗p < 0.01, ∗∗∗p < 0.001. Data represented as mean ± SEM.

To study the impact of Klf5 ablation in adaptive remodeling of muscle *in vivo*, mice were intravenously injected with myotropic AAV (AAVMyo) expressing Klf5-targeting shRNA. 4 weeks post-injection, mice received access to running wheels for 6 weeks. 10 weeks post-injection, knockdown efficiency was evaluated at the end of the experiment in *m. gastrocnemius* (GAS) (KD-efficiency = 66%; Figure 3A) via qPCR. Klf5 ablation did not affect body weight of the animals (data not shown). KLF5KD mice exhibited significant reduction in voluntary running activity after 6 weeks of the intervention (Figure 3B) and displayed reduced running performance in an exercise-capacity test (Figure 3C) while increasing blood lactate significantly (Figure 3D), accompanied by an elevated respiratory exchange ratio during the running bout (Figure 3E). All of these findings point towards decreased aerobic capacity. Following dissection, we observed variations in heart weight between the SCR and KLF5KD groups. KLF5KD mice presented significant cardiac hypertrophy (Figure 3F), which negatively correlated with running distance (Figure 3G), and presented signs of dilated cardiomyopathy (Figure 3H). The role of Klf5 in hypertrophic cardiomyopathy was previously studied by Drosatos et al. [34]. Klf5 is a major player in diabetic and dilated cardiomyopathy, however the rapid onset of this pathology (10 weeks post-injection) were in stark contrast to the mild cardiac hypertrophy observed in heart-specific Klf5 knockout mice at 10 months of age [35], and opposite to the observation that inhibition of Klf5 seems to have protective effects in ischemic cardiomyopathy [36]. Thus, impaired cardiac function due to Klf5 knockdown hampers the interpretation of the results, particularly those obtained in exercise tests.

### 3.4 Klf5 ablation impairs lipid utilization after an acute bout of exercise

**Figure 4:**
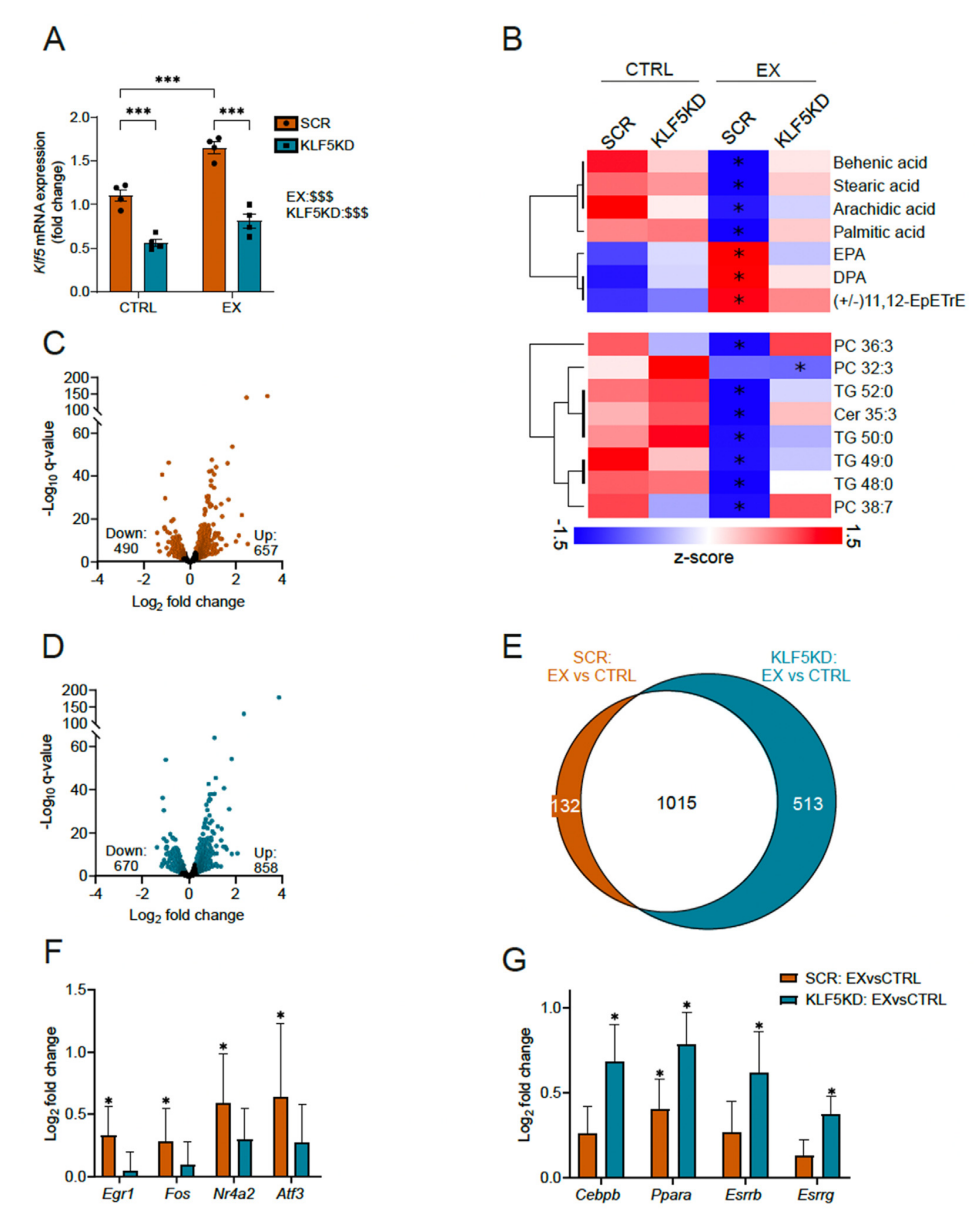
**Klf5 ablation impairs lipid utilization after an acute bout of exercise.** **A)** Relative Klf5 mRNA expression in injected *m. gastrocnemius* (n = 4, 4, 4, 4). **B)** Overview of significant differentially detected lipids in negative mode (upper heatmap) and positive mode (lower). Stars mark significant change compared to the respective CTRL group. **C)** Differentially detected genes in SCR: EX vs CTRL (p < 0.05; FC = 1.2; n = 4, 4). **D)** Differentially detected genes in KLF5KD: EX vs CTRL (p < 0.05; FC = 1.2; n = 4, 4). **E)** Overlap of the differential expression analysis between SCR: EX vs CTRL and KLF5KD: EX vs CTRL. **F)** Transcription factors found significantly upregulated by exercise in SCR with no upregulation in the KLF5KD post-exercise. **G)** Transcription factors found significantly upregulated in KLF5KD post-exercise with absent/blunted regulation in SCR. Statistical test: two-way ANOVA (repeated measures) followed by Sidak's multiple comparisons 2 groups: A. In B) significance is indicated based on the lipidomic analysis in comparison to the respective CTRL group. In F, G) Significance is indicated based on the transcriptomic analysis in comparison to the respective CTRL group. Data represented as mean ± SEM.

To circumvent the hypertrophic cardiomyopathy associated with Klf5 ablation in the systemic modulation experiment in the heart, we adopted an alternative strategy to specifically assess the molecular consequences of Klf5 loss in skeletal muscle. To do so, given that current myotropic AAV capsids used for systemic delivery also transduce cardiac tissue, we aimed for a localized knockdown within the gastrocnemius muscle. To this end, we performed intramuscular injections and compared the targeted muscle to the contralateral, untreated gastrocnemius muscle of the same animal as an internal control. In order to prevent unintended viral spread to adjacent muscles, we employed the wild-type AAV9 capsid, as AAVMyo has previously been reported to disseminate beyond the injection site under similar conditions [37]. KLF5 ablation in one leg of the animal was achieved through the intramuscular (i.m.) administration of AAV9 carrying Klf5-targeting shRNA into the gastrocnemius muscle (GAS), with the contralateral leg receiving a control virus containing scrambled shRNA (SCR). This approach circumvents potential inter-animal variations in running distance and training intensity, given that the comparison between Klf5 knockdown and control muscle is made in the same animal. To assess the effect of Klf5 ablation in an acute exercise context, animals performed a maximum capacity test and tissue was harvested 3h post-exercise. Knockdown efficiency in the shRNA-injected GAS of the non-running control (CTRL) group was 49%, in the same range (52%) as that in animals undergoing a single bout of maximal exercise on a treadmill (EX) (Figure 4A). Given that AAV9 delivery can result in transduction of multiple cell types within skeletal muscle, and considering that KLF5 is expressed in both fibroadipogenic progenitors (FAPs) and muscle fibers [38], we additionally performed intravenous injections using AAVMyo encoding miRNAs against KLF5 under the control of the dMCK promoter [39,40]. This strategy was chosen to ensure that the observed knockdown reflects reduced KLF5 levels specifically in muscle fibers, rather than in mononucleated muscle-resident cells. With this approach, we observed a reduction in KLF5 expression that was comparable to that seen with the AAV9-based delivery (Fig. S2). These findings indicate that the primary source of KLF5 knockdown is indeed the muscle fiber. Unfortunately, the dMCK promoter is known to drive expression much more potently in glycolytic compared to oxidative fibers, which we confirmed when assessing the knockdown efficiency in the soleus muscle (Fig. S2C). All further experiments were therefore performed with the AAV9-Klf5 shRNA samples to avoid confounding effects of the dMCK-promoter activity variations in the different muscle fiber types.

Based on the *in vitro* and *in vivo* data implying a role for Klf5 in muscle lipid homeostasis, we performed lipidome analysis of the injected muscles. A significant blunting of the exercise effect, thus the difference between EX and CTRL groups, on different lipid species was observed, with only one change (PC 32:3) in the KLF5KD compared to the 15 changes in the SCR animals (Figure 4B, Supplementary Data set KLF5_Manuscript_Lipidomics.xlsx). Specifically, while the exercise-intervention depleted triglycerides and free fatty acids including behenic, stearic and palmitic acid in the SCR-condition, KLF5KD did not display a similar reduction.

Fatty acids are the major fuel source for skeletal muscle during submaximal exercise. Lipids can either be recruited from intramuscular triacylglycerols (IMTGs) or can be taken up from the bloodstream in VLDLs or as free fatty acids [41]. To elucidate the mechanistic underpinnings for this difference in lipid utilization, bulk RNAseq of the muscles was performed (Supplementary Data set KLF5_Manuscript_RNA-Seq.xlsx). Analysis of the GAS transcriptome 3h post-exercise revealed 1147 differentially expressed genes (DEG) in the comparison of the SCR muscles of sedentary and exercised animals (Figure 4C; upregulated: 657, downregulated: 490), and 1528 DEG in the corresponding KLF5KD muscles (Figure 4D; upregulated: 858, downregulated: 670). Of these, 132 and 513 genes were exclusively regulated in the SCR and KLF5KD GAS, respectively (Figure 4E). Interestingly, Klf5 ablation increased the number of upregulated genes compared to the control, suggesting a potential suppressive function, secondary effects or compensatory processes. Among the genes exclusively upregulated by exercise in the control group are classical early stress response genes including Egr1, Fos, Nr4a2, and Atf3 (Figure 4F). The blunting of these genes upon Klf5 ablation points towards a potential recursive, upstream role of this transcription factor in the induction of the stress response of muscle reacting to contractile activity. Conversely, we observed an increase of transcription factors involved in lipid metabolism, especially fatty acid beta oxidation, in the KLF5KD muscles, including Cebpb, Ppara, Esrrb and Esrrg (Figure 4G), which seems paradoxical in light of reduced lipid utilization (Figure 4B).

### 3.5 Loss of Klf5 impairs training-mediated increase in proteins involved in fatty acid biosynthetic processes

**Figure 5:**
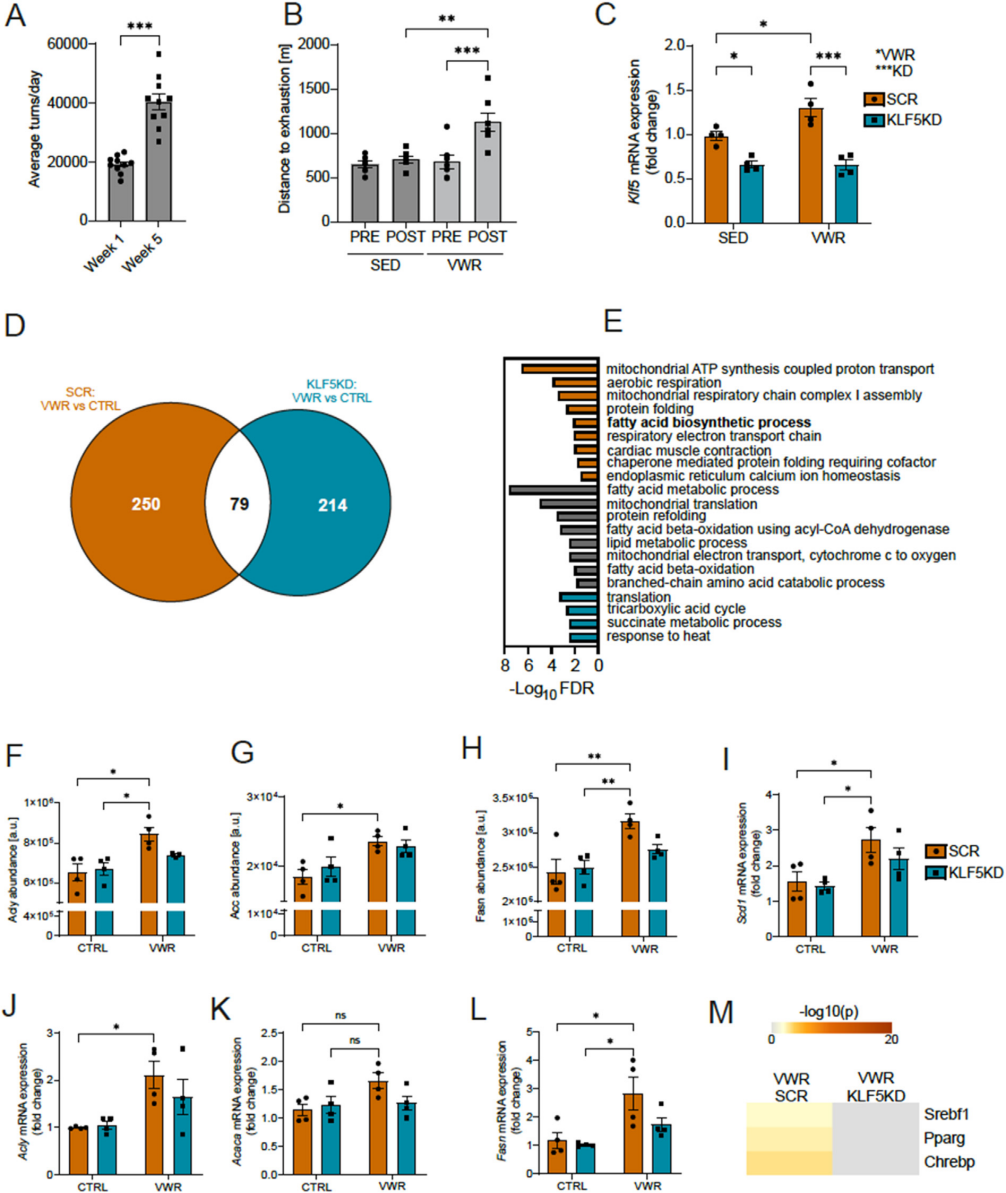
**Loss of Klf5 impairs exercise-mediated increase in proteins involved in fatty acid biosynthetic processes.** **A)** Increase in average turns/day for each mouse with access to running wheels in the 1st and 5th week. **B)** Maximum capacity test of SED and VWR mice at the end of the VWR intervention. **C)** Relative Klf5 mRNA expression in SED and VWR in GAS. **D)** Overlap of the differentially expressed proteins between SCR: EX vs CTRL and KLF5KD: EX vs CTRL. **E)** Overlap of the biological processes of the upregulated proteins in SCR: EX vs CTRL and KLF5KD: EX vs CTRL. Orange indicates GO-terms exclusively found in SCR, grey indicates shared terms, cyan for exclusively found in KLF5KD. **F)** Relative Acly protein abundance in GAS (n = 4, 4, 4, 4). **G)** Relative Acc protein abundance in GAS (n = 4, 4, 4, 4). H) Relative Fasn protein abundance in GAS (n = 4, 4, 4, 4). I) Relative Scd1 mRNA expression. J) Relative Acly mRNA expression. K) Relative Acaca mRNA expression. L) Relative Fasn mRNA expression. M) Transcription factor activity prediction of differentially expressed genes in SCR: EX vs CTRL and KLF5KD: EX vs CTRL. Statistical test: two-way ANOVA (repeated measures) followed by Sidak's multiple comparisons 2 groups: B, C, I, J, K, L; students t-test: A, F, G, H ∗p < 0.05, ∗∗p < 0.01, ∗∗∗p < 0.001. Data represented as mean ± SEM.

The *in vitro* and *in vivo* experiments revealed that the transient induction of Klf5 in myofibers after an acute endurance exercise bout is linked to modulation of lipid homeostasis, most prominently the use of fatty acids as energy substrates. We next investigated the consequence of Klf5 overexpression on chronic, long-term training adaptations. We used the same experimental setup (contralateral leg injections of Klf5-targeting shRNA and scrambled control shRNA in AAV vectors) to reduce Klf5 levels in one GAS per mouse. Six weeks of voluntary wheel running (VWR) resulted in a trained phenotype, underlined by an increase in running wheel distance run per day over time (Figure 5A), as well as improved endurance performance in a maximum capacity test on a treadmill (Figure 5B). In this setting, knockdown efficiency is 43% for the sedentary and 49% for the VWR-group (Figure 5C). Moreover, an increase in baseline Klf5 levels was detected in trained GAS muscles (Fig. 5C). Proteomic analysis of the trained muscles resulted in 329 differentially expressed proteins (221 up- and 108 downregulated) in SCR and 293 differentially abundant proteins in KLF5KD (222 up- and 71 downregulated). A comparison of the two conditions revealed only a small overlap of 79 proteins (Figure 5D, Supplementary Data set KLF5_Manuscript_Proteomics.xlsx). Functional analysis of upregulated proteins in each condition lead to a clear stratification of GO terms. The overlap between SCR and KLF5KD is dominated by proteins contributing to fatty acid beta oxidation, implying that Klf5 is dispensable for the corresponding training adaptations. Similarly, some terms describing mitochondrial function are found in the overlap with the KLF5KD muscles. Notably, the SCR only group shows further enrichment in terms related to mitochondrial respiration, and exclusive occurrence of muscle contractility and calcium homeostasis. To assess whether KLF5 ablation is associated with mitochondrial respiratory chain dysregulation, we examined the abundance of mitochondrial complex subunits identified in the proteomic analysis (Fig. S3). No significant differences were observed in the levels of any of the mitochondrial complex subunits, suggesting that KLF5 loss does not directly affect the composition of the respiratory chain. Intriguingly, in contrast to the catabolic terms of lipid metabolism, “fatty acid biosynthetic process” is likewise only detected in SCR, but not KLF5KD muscles, indicating a requirement for muscle Klf5 for these training-related process. Loss of Klf5 leads to a compensatory increase in proteins included in the GO terms encompassing tricarboxylic acid cycle (TCA) cycle, succinate metabolism and proteostatic stress (“response to heat”) (Figure 5E). This divergence of Klf5 dependence for fatty acid metabolism (Klf5-independent training adaptation) and lipid biosynthesis (Klf5-dependent training adaptation) is surprising in light of the results obtained after an acute exercise bout, in which the former, namely catabolism, is controlled by Klf5. Regulated proteins that are contributing to the significant enrichment of the term “fatty acid biosynthetic process” include key players in palmitate synthesis from acetyl-CoA such as Acly, Acaca, and Fasn, all of which exhibited increased levels in the CTRL leg, but not upon Klf5 ablation (Figure 5F–H). These changes in protein level were reflected in the induction of the respective mRNAs (Figure 5J–L). Moreover, higher transcript levels of Scd1 were likewise found (Figure 5I), mirroring the *in vitro* overexpression results of Klf5 in C2C12 myotubes (Figure 2E). Motif enrichment analysis (TTRUST) on the upregulated proteins predicted increased activity of the transcription factors Srebf1, Pparγ and Chrebp in SCR, which was absent in the KLF5KD condition (Figure 5M). These three transcription factors have clear functions in the regulation of lipogenesis [[42], [43], [44]]. Corroborating these changes in lipid metabolism, ablation of Klf5 via the dMCK-miRNA model resulted in altered Srebf1 expression in quadriceps muscle and both gastrocnemius subregions (white and red). In contrast, the training-dependent increase in Srebf1 was restricted to the white gastrocnemius and was abolished in KLF5KD samples (Figure S4 A-F).

### 3.6 Ablation of Klf5 leads to accumulation of short-chain acylcarnitines, palmitate and ceramides

**Figure 6:**
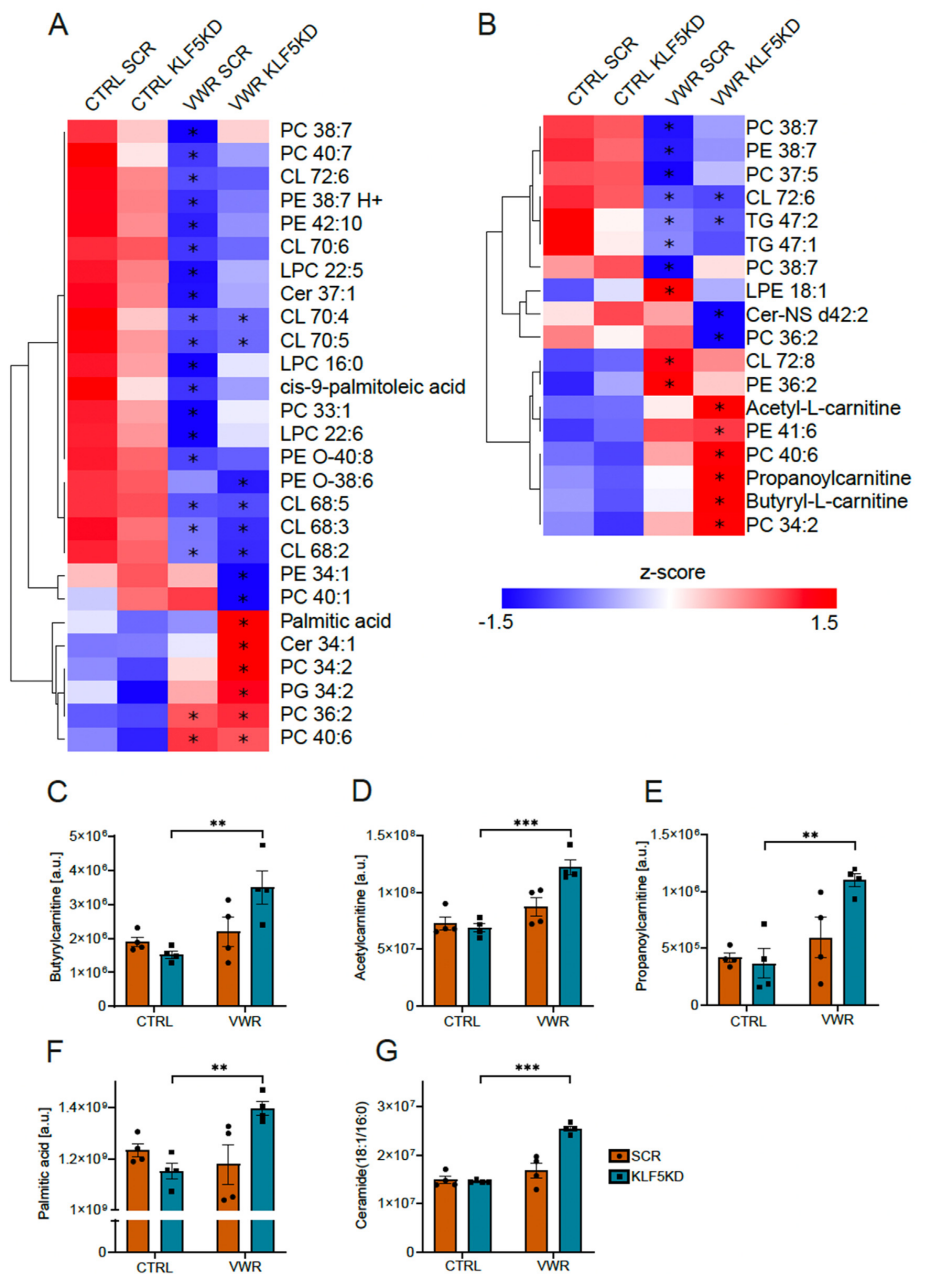
**Ablation of Klf5 accumulates short-chain acylcarnitines, palmitate and ceramides.** **A)** Heatmap of significantly detected lipids between CTRL and VWR in negative mode. Stars mark significant change compared to the respective CTRL group. **B)** Heatmap of significantly detected lipids between CTRL and VWR in positive mode. Stars mark significant change compared to the respective CTRL group. **C)** Detection levels of Acetylcarnitine, **D)** Propanoylcarnitine, **E)** Butyrylcarnitine, **F)** Palmitic acid and **G)** Ceramide (34:1)-levels in injected GAS (arbitrary units, n = 4, 4, 4, 4). Statistical test: student's t-test (based on lipidomic analysis) C-G. In A, B) significance is indicated based on the lipidomic analysis in comparison to the respective CTRL group. Data represented as mean ± SEM.∗p < 0.05, ∗∗p < 0.01, ∗∗∗p < 0.001.

Based on the proteomic data, GO analysis and regulatory prediction, a muscle Klf5-dependent strong modulation of lipid homeostasis, and more specifically lipogenesis, by long-term training could be inferred. Thus, to assess whether these processes are indeed affected, a lipidomic analysis was performed. This analysis revealed 45 lipids (27 in negative mode, 18 in positive mode) with differences in abundance between the sedentary and trained muscles of both conditions (Figure 6A,B). For example, VWR had a major effect on different cardiolipin (CL)-species. Mature CL (72:8) levels were increased while intermediate CLs (CL 72:6, CL 70:6, CL 70:4, CL 70:5, CL 68:5, CL68:3, CL68:2) were less abundant in trained muscle. Of note, in contrast to the similar training-mediated regulation of intermediate CLs in SCR and Klf5KD muscles, the elevation of mature CL was blunted in the latter condition. Moreover, only the Klf5KD lead to an increase in short-chain acyl-carnitines, namely acetyl-l-carnitine, propanoylcarnitine and butyryl-carnitine (Figure 6C,D, E) in the trained context. Likewise, only the Klf5KD resulted in a training-dependent increase in the saturated palmitic acid and ceramide (18:1/16:0) (Figure 6F,G). These data corroborate the intimate involvement of Klf5 in the control of training-linked lipid remodeling in skeletal muscle.

## 4 Discussion

Skeletal muscle reacts to an acute bout of exercise with a strong transcriptional response that, at least in part, differs between an untrained and a trained muscle [45]. Klf5 is one of the transcription factors that is transiently expressed in the early hours following an acute exercise bout in mice and humans. We have now unraveled the function of muscle Klf5 in an acute endurance exercise bout and for steady-state long-term training adaptations in mice *in vivo*. In essence, a strong role of Klf5 in modulating lipid homeostasis has been discovered, with surprisingly divergent outcomes in the acute and chronic settings. Importantly, the regulation of the corresponding genes could be corroborated by overexpression of Klf5 *in vitro* in C2C12 myotubes, and ChIP-seq data obtained previously in the same cell line.

First, shRNA-mediated reduction of Klf5 in an acute exercise context leads to an impaired utilization of lipids, as evidenced by the lack of reduction in saturated triglycerides and free fatty acids in the exercised gastrocnemius muscles. Intriguingly, at the same time, Klf5KD increased the expression of Cebpb, Ppara, Esrrb and Essrg. These transcription factors are potent regulators of fatty acid beta-oxidation but seem to lack the capability to modulate this gene program in the absence of Klf5. It thus is unclear whether the induction of gene expression of these transcription factors is a secondary compensatory, albeit unsuccessful mechanism to cope with the impaired lipid oxidation. Intriguingly, many other transcription factors are also among the differentially regulated genes in the Kl5KD, including those involved in the immediate early stress gene regulation, postulated to be upstream of Klf5 in cancer and other cells. Thus, a much more complex regulatory network, including feedback mechanisms, seems to center on Klf5. Zhao et al. previously described “stripe” transcription factors, a family of universal TFs binding to GC-rich sequences, co-binding/recruiting other TFs to their respective promoters [46]. Multiple members of the Klf-family have been postulated to be such stripe transcription factors [12], which, based on our data, could include Klf5 in skeletal muscle. This potential role in helping to recruit other TFs to their respective promoter might be implied by the altered TF activity prediction of Foxo1, Nfkb1 and Cebpb, all of which are less active in the KLF5KD, and that have previously been described to interact with Klf5 [10,34,40].

The mechanistic underpinnings of long-term training adaptation are still only poorly understood. It however has been demonstrated that even transcriptional regulators that are only transiently induced after an acute endurance exercise bout such as PGC-1α are indispensable for a normal training response [45]. We therefore investigated the function of Klf5, which shows similar regulatory kinetics as PGC-1α, in the context of chronic training. Intriguingly, the persistent knockdown of Klf5 evoked an adaptive response that differed from the short-term effects after an acute exercise bout. In contrast to the mitigation of fatty acid beta oxidation in the latter context, trained muscles with Klf5 knockdown exhibited a broad dysregulation of various lipid species, most notably related to lipogenic pathways. Lipogenesis is an important process in endurance training adaptation, resulting in increased storage of neutral triglycerides as energy substrates, as well as a shift towards lipid species with beneficial properties, for example CL that is instrumental for mitochondrial function. These specific adaptations provide an explanation for the “athletes paradox”, in which an increase in intramyocellular lipids in trained athletes and type 2 diabetic patients has been described [47]. The relative amount of lipid species and the localization in the muscle cell are clearly different in these populations, combined with a much higher turnover rates in athletes compared to type 2 diabetic patients [48]. We now demonstrate that in the absence of functional levels of muscle Klf5, the normal training adaptation in terms of lipid homeostasis is significantly impaired. Importantly, a shift from “good” lipid species, such as mature CL, to potentially pathological lipid species, including the saturated palmitic acid and ceramide, is observed. Genes affected by the KLF5KD include Acly and Scd1, which are both essential proteins in lipid biosynthesis [49]. Acly ablation leads to reduced cardiolipin synthesis and therefore decreased oxygen consumption [50]. Overexpression of Scd1 in skeletal muscle and heart results in increased exercise capacity and abundance of polyunsaturated fatty acids (PUFAs) [51]. The regulation of Scd1 by Klf5 post-exercise could therefore contribute to the increase in PUFAs in trained muscle [26,27]. Inversely, the increase in short-chain acyl-carnitines, palmitate and ceramide in the trained KLF5KD muscles might indicate an unfavorable context, evoked by the inability to process lipid species in a normal manner. Short chain acetyl-carnitines (ACs), especially C2-, C3- and C4-ACs can be the product of branched chain amino acid catabolism or peroxisomal lipid oxidation [39]. As short-chain carnitines do not need to be actively transported into the mitochondria, this could point towards a reduced capacity of mitochondria to import medium to long-chain acylcarnitines [39,40]. Moreover, the incomplete metabolism of acyl-carnitines to acetyl-carnitine has been associated with a disconnect between fatty acid transport and mitochondrial import, fatty acid beta oxidation, the TCA cycle and mitochondrial respiration, as observed in insulin-resistant and diabetic muscle [52]. Similarly, an accumulation of saturated fatty acids such as palmitate, or of sphingolipids, in particular ceramide, have been associated with the development of skeletal muscle insulin resistance [53], as well as disease progression in cardiomyopathy or coronary artery disease [54]. Whether the changes elicited by KLF5KD in trained muscle indeed result in a pathological outcome remains to be determined.

All of these alterations are accompanied by a reduction in the predicted activity of important regulators of this process, including Pparγ and Srebf1, an interaction which has previously been observed in other tissues [55]. In fact, Klf5 co-regulation of Srebf1 in the control of lipid biogenesis has been found in squamous and prostate cancer [55,56]. We now show similar Klf5-dependent processes in the physiological context of skeletal muscle adaptation to training.

Limitations: The experiments presented in this study were primarily conducted in the gastrocnemius muscle, which consists predominantly of glycolytic type 2× and type 2B fibers. For future investigations, it would be of considerable interest to assess the role of KLF5 in additional muscle groups, including those with a more oxidative, slow-twitch fiber composition such as the soleus. Given the distinct metabolic profile and higher capacity for lipid utilization in the soleus, it may respond differently to KLF5 modulation, and such studies could provide further insight into fiber type-specific functions of KLF5. In addition, time point-specific analyses of KLF5 activation both following acute exercise but also during the chronic training adaptation in the first weeks of an exercise-regimen warrant further investigations. In the current study, our analysis was restricted to sedentary animals subjected to an acute exercise stimulus and to trained animals in the absence of acute stimulation. However, prior studies have demonstrated that both the magnitude and timing of gene expression responses differ markedly between naïve and trained muscle, with trained muscle exhibiting a more rapid transcriptional response to acute exercise [45]. These findings raise the question of whether KLF5 is similarly subject to accelerated or altered activation kinetics in the trained state, a topic that should be addressed in future work.

In summary, we demonstrate that the transcription factor Klf5 exerts important functions in short-term acute exercise response and long-term endurance training adaptation. Even though having primarily been implicated in the immediate early stress gene response downstream of Egr-1, we found a very specific role for Klf5 in controlling lipid homeostasis by increasing fatty acid beta oxidation in an acute exercise bout, and by modulating lipogenesis and lipid species conversion in the chronic training contexts, respectively. Based on these findings, it will be interesting to investigate whether specific targeting of Klf5 in pathological contexts, for example insulin resistance or type 2 diabetes, is sufficient to rectify lipotoxicity and improve lipid oxidation. If so, the results obtained in the exercise contexts will have important clinical implications for the prevention and/or therapy of metabolic diseases.

## CRediT authorship contribution statement

**Konstantin Schneider-Heieck:** Writing – review & editing, Writing – original draft, Visualization, Validation, Methodology, Investigation, Formal analysis, Conceptualization. **Joaquín Pérez-Schindler:** Writing – review & editing, Visualization, Supervision, Methodology, Investigation, Conceptualization. **Jonas Blatter:** Methodology, Investigation, Formal analysis, Conceptualization. **Laura M. de Smalen:** Methodology, Investigation, Formal analysis, Conceptualization. **Wandrille Duchemin:** Software, Resources, Methodology, Formal analysis, Data curation. **Stefan A. Steurer:** Resources, Methodology, Investigation. **Bettina Karrer-Cardel:** Resources, Methodology, Investigation. **Danilo Ritz:** Resources, Methodology, Investigation, Formal analysis, Data curation. **Christoph Handschin:** Writing – review & editing, Writing – original draft, Visualization, Validation, Supervision, Project administration, Funding acquisition, Formal analysis, Conceptualization.

## Declaration of generative AI in scientific writing

During the preparation of this work the author(s) used ChatGPT 4o in order to improve the readability and language of the manuscript. After using this tool/service, the author(s) reviewed and edited the content as needed and take(s) full responsibility for the content of the published article.

## Funding sources

This research was supported by the Swiss National Science Foundation (Schweizerischer Nationalfonds zur Förderung der Wissenschaftlichen Forschung, grant CRSII5_209252), the Biozentrum, and the University of Basel.

## Declaration of competing interest

The authors declare the following financial interests/personal relationships which may be considered as potential competing interests:

Christoph Handschin reports financial support was provided by Swiss National Science Foundation. If there are other authors, they declare that they have no known competing financial interests or personal relationships that could have appeared to influence the work reported in this paper.

## Data Availability

Data will be made available on request.
