# Supplementary material for: Krüppel-like factor 5 remodels lipid metabolism in exercised skeletal muscle
Source: Mol Metab. 2025 Apr 16;96:102154. doi: 10.1016/j.molmet.2025.102154 (PMC12060515; doi:10.1016/j.molmet.2025.102154)

Supplemental Figure Legends

Figure S1: Metabolic impact of KLF5 overexpression on C2C12 myotubes.

A) Oxygen consumption rate during the mitochondrial stress test. **B)** Extracellular acidification rate during mitochondrial stress test assay. **C)** Oxygen consumption rate during the glycolytic rate assay. **D)** ECAR during glycolytic rate assay. Injected compounds are displayed on the lines – each measuring time point is 2min. **E)** Fold change of the baseline ECAR compared to the respective control in the glycolytic rate assay. **F)** Fold change of glycolytic proton efflux rate. Each dot in the graphs represents an independent experiment. Statistical test: E, F; student's t-test: A, B, C, F. *p<0.05, **p<0.01, ***p<0.001. Data represented as mean ± SEM.

Figure S2: Muscle-specific expression of KLF5-targeting miRNA shows ablation of KLF5 in muscle fibers, with a preference for glycolytic fibers.

A) Relative Klf5 mRNA expression in the white (glycolytic) part of the *m. gastrocnemius* (n = 6, 6). **B)** Relative Klf5 mRNA expression in the *m. quadriceps femoris* (n = 6, 6). **C)** Relative Klf5 mRNA expression in the *m. soleus* (n = 6, 6). Statistical test: student's t-test: A, B, C. *p<0.05, **p<0.01, ***p<0.001. Data represented as mean ± SEM.

Figure S3: Abundance of mitochondrial complex subunits detected in the proteomic analysis.

Fold change of the proteins associated with **A)** Complex I, **B)** Complex II, **C)** Complex III, **D)** Complex IV, **E)** Complex V. Data represented as mean ± SEM.

Figure S4: Muscle-specific expression of KLF5-targeting miRNA shows dysregulation of Srebf1 expression, with training-induced changes in Srebf1 expression being absent in the glycolytic part of the gastrocnemius muscle.

A) Relative Klf5 mRNA expression in the m. quadriceps femoris (n = 6, 6). **B)** Relative Klf5 mRNA expression in the white (glycolytic) part of the m. gastrocnemius (n = 6, 6). **C)** Relative Klf5 mRNA expression in the m. soleus (n = 6, 6). Srebf1 expression in CTRL and VWR-condition in **D)** quadriceps femoris, **E)** glycolytic (white) part of the m. gastrocnemius, **F)** oxidative (red) part of the m. gastrocnemius. Statistical test: two-way ANOVA (repeated measures) followed by Sidak's multiple comparisons 2 groups: A, B, C, D, E, F. *p<0.05, **p<0.01, ***p<0.001. Data represented as mean ± SEM.

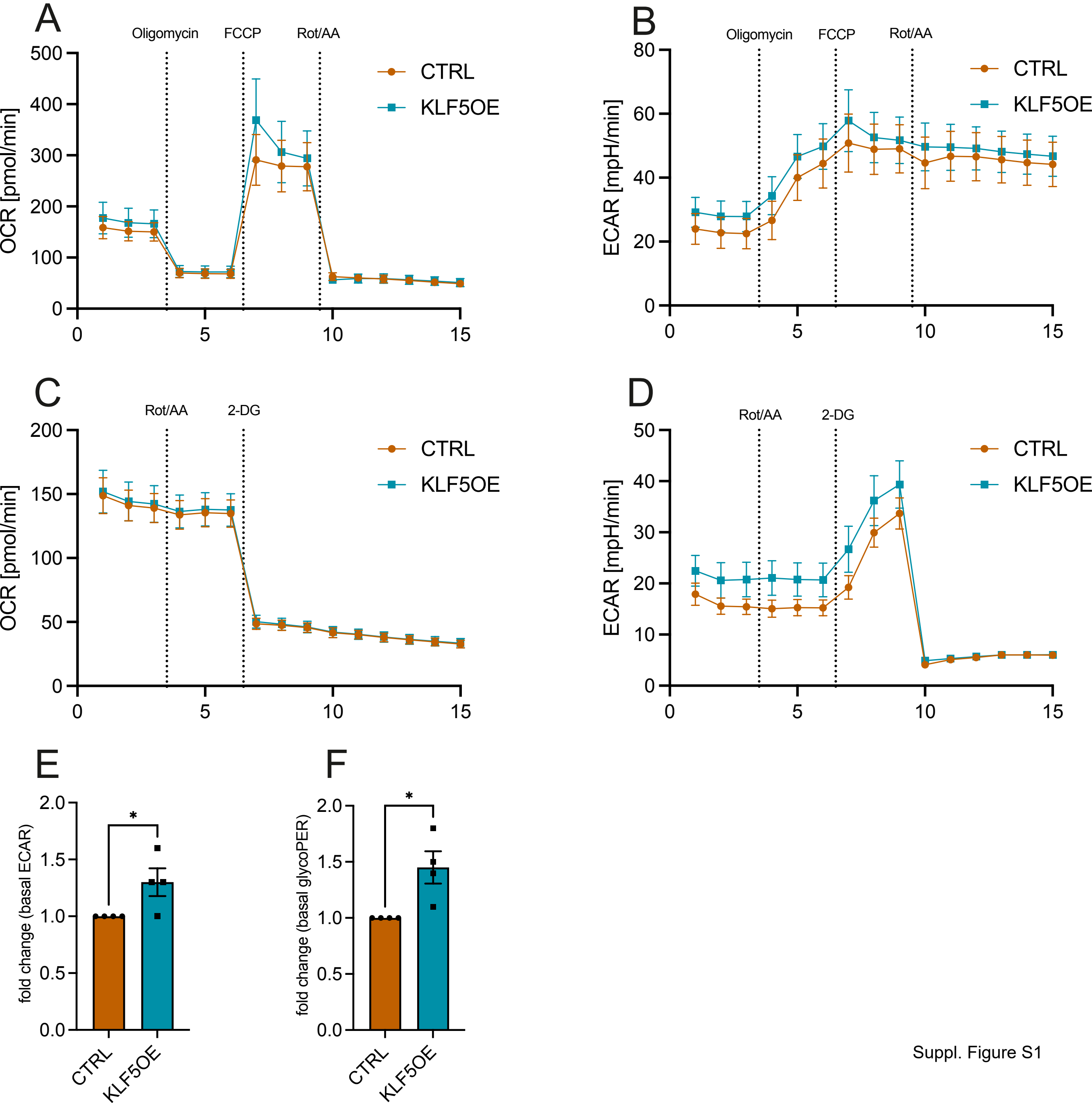

Suppl. Figure S1

Suppl. Figure S2

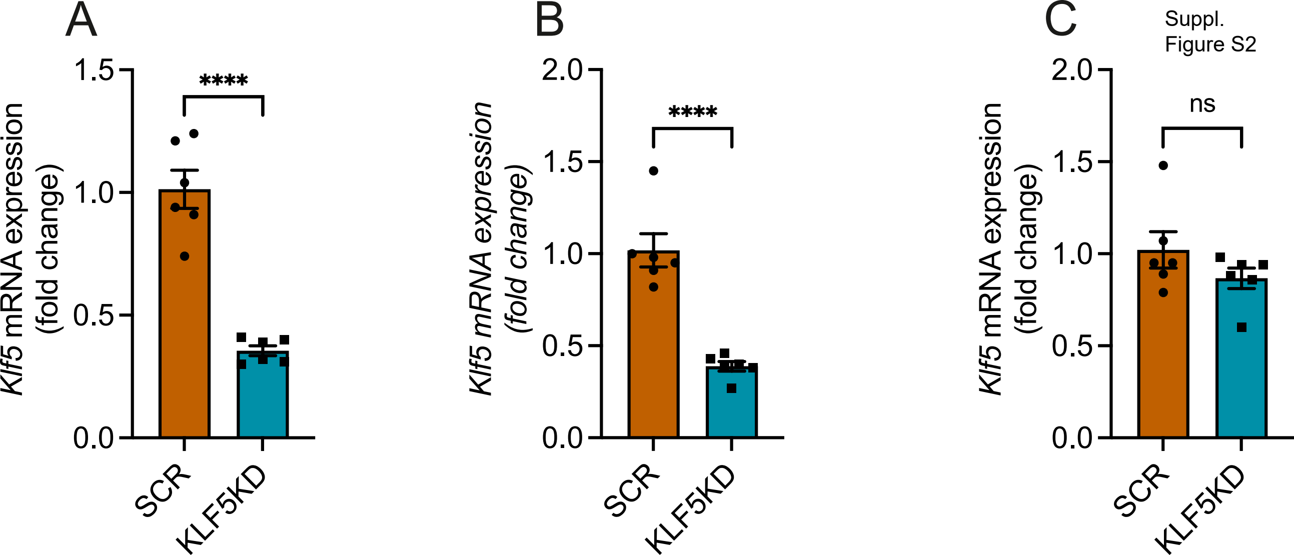

Suppl. Figure S3

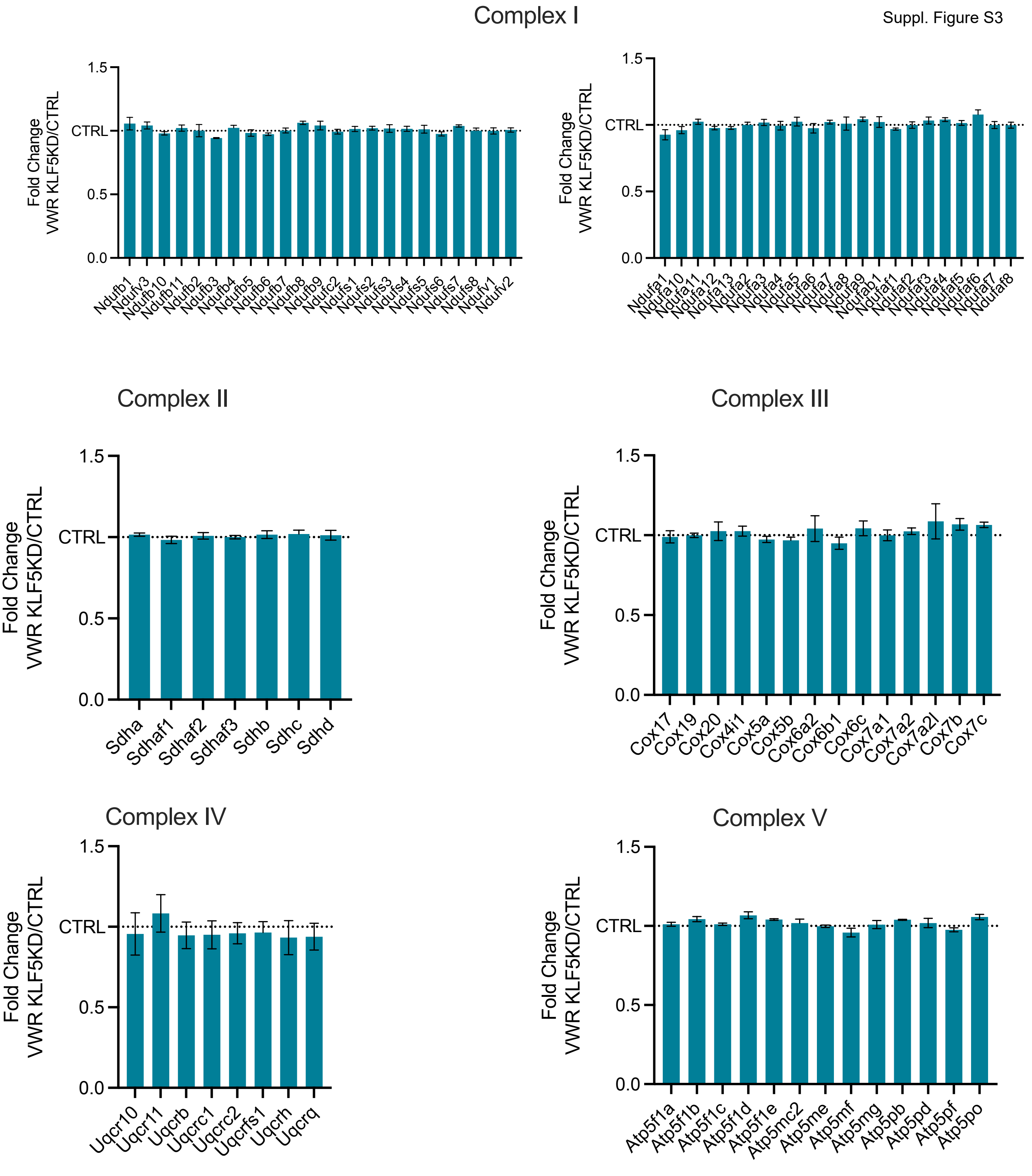

Suppl. Figure S4

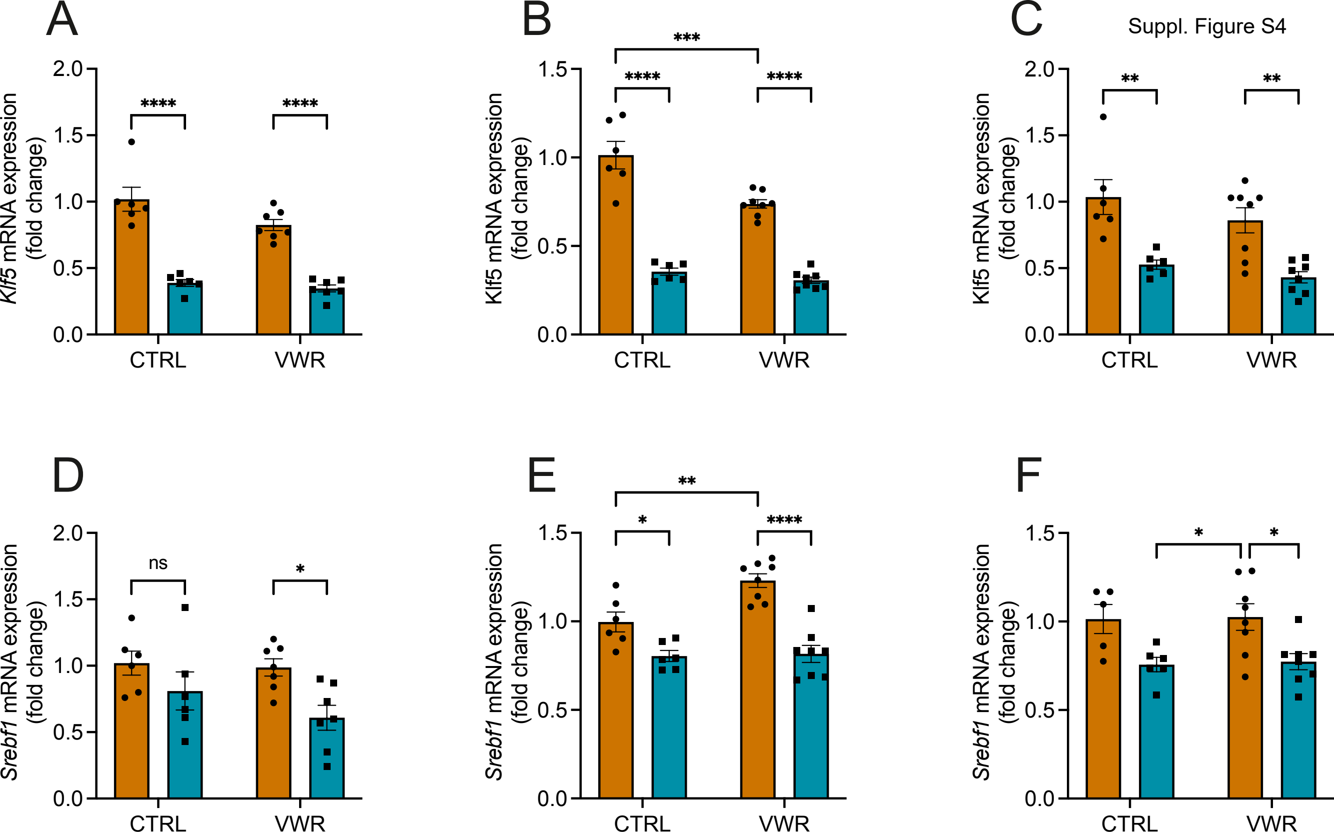

Supplement: Multimedia component 1 [file mmc1.pdf]
